# Cross-Education Effects After Submaximal and Supramaximal Accentuated Eccentric Loading on Lean Mass and Function in Women

**DOI:** 10.3390/jfmk11010063

**Published:** 2026-01-31

**Authors:** Sergio Maroto-Izquierdo, Miguel Lauría-Martínez, Kayvan Khoramipour, Irati Jauregui-Fajardo, Paula Redondo-Delgado, José Antonio de Paz, David García-López

**Affiliations:** 1 i+HeALTH Strategic Research Group, European University Miguel de Cervantes, 47012 Valladolid, Spain; 2 Proporción A, Applied Sports Science Center, 47015 Valladolid, Spain; mlauria@uemc.es; 3 Faculty of Health Science, Department of Health Science, European University Miguel de Cervantes, 47012 Valladolid, Spain; 4 Institute of Biomedicine, University of León, 24401 León, Spain

**Keywords:** eccentric-overload, strength, hypertrophy, muscle power, vertical jump, muscle endurance, female

## Abstract

**Objective:** This study compared the effects of submaximal and supramaximal accentuated eccentric loading (AEL) on lean mass and function in the trained (TL) and contralateral non-trained (NTL) legs of women. **Methods:** Twenty recreationally trained women were randomly assigned to submaximal (90% 1-RM) or supramaximal (120% 1-RM) AEL leg press training (2/week, 10 weeks, 4 sets of 8 repetitions) with 30% 1-RM concentric loading. Total thigh lean mass (TTLM), unilateral leg press 1-RM, mechanical power at 40% (P40), 60% (P60), and 80% (P80) of 1-RM, unilateral countermovement (CMJ) and drop jump (DJ) height, and muscle endurance (XRM) were assessed for each leg before and after intervention. **Results:** Regarding the TL, the submaximal group showed significant (*p* < 0.05) increases in 1-RM, P40, CMJ, and DJ, while the supramaximal group showed increased TTLM, 1-RM, P40, P60, and XRM. No significant differences were observed between groups. In the NTL, both groups showed significant increases in 1-RM and P40. Additionally, the submaximal group demonstrated improvements in P60, while the supramaximal group showed significant increases in both P60 and P80, and in TTLM. TL and NTL changes correlated significantly for 1-RM, CMJ, and TTLM. However, TL and NTL changes differed significantly for 1-RM and P40 in the submaximal group and for TTLM in the supramaximal group. **Conclusions**: Submaximal and supramaximal AEL resulted in similar neuromuscular improvements in both TL and NTL in women. Supramaximal loading provided additional benefits in mechanical power lean mass, while submaximal loading improved explosive performance. Supramaximal loading may not be necessary for active women.

## 1. Introduction

Resistance training (RT) is the cornerstone for improving muscle strength, function, and mass in both healthy and clinical populations [1]. Training intensity during RT is a major factor in these adaptations [2]. Conventional RT includes both concentric (lifting) and eccentric (lowering) phases, with loads usually set relative to the maximal concentric strength [3]. However, because muscles can generate greater force during lengthening, eccentric muscle contraction is often underloaded, limiting neuromuscular adaptations [4,5].

Accentuated eccentric loading (AEL) addresses this limitation by prescribing eccentric loads that exceed those lifted during the concentric phase [6,7]. Electric-driven devices [8,9], weight releasers [10,11], and flywheel technology [12,13] facilitate eccentric loads 25–40% higher than concentric loads. AEL has been shown to improve muscle strength, power, and hypertrophy more effectively than traditional RT [14,15,16,17]. Although there is some controversy regarding the optimal magnitude of eccentric overload [6], recent evidence suggests that submaximal eccentric loading may be sufficient to induce neuromuscular adaptations comparable to those achieved with supramaximal loading in healthy men [18]. However, further research is needed to clarify which eccentric loading strategies are most effective in maximising training results in different populations.

A notable advantage of AEL is the enhancement of the cross-education (CE) effect, in which unilateral resistance exercise training improves strength in both the trained ipsilateral limb and the homologous muscles in the non-trained contralateral limb [19,20]. It is widely known that eccentric contractions elicit unique neural patterns that may facilitate CE [5,7,21]. When eccentric contractions are preceded by a concentric one, the stretch-shortening cycle is engaged, thereby amplifying the neural stimulus and resulting in movement patterns that closely resemble functional tasks [22]. Consequently, AEL, by integrating concentric and eccentric phases, may further enhance the CE effect through the recruitment of global stabilising musculature and the promotion of task-specific neural adaptations [22,23].

However, the optimal eccentric load to maximise training-induced adaptations in both trained and untrained limbs remains unclear. While supramaximal loads (>100% concentric 1-RM) are common, some studies report similar adaptations with submaximal loads [18,24,25,26]. Concerns regarding the potential risk of injury and limited practical benefits have led to further questions on the need for supramaximal loads [24]. Furthermore, given potential gender differences in neuromuscular adaptation [27], it is crucial to determine whether previous findings in men regarding strength and hypertrophy are generalisable to women [18,22,28]. Thus, the effects of submaximal versus supramaximal AEL on CE and neuromuscular adaptations in women are still largely unknown. Therefore, this study compares the effects of submaximal and supramaximal AEL on lean mass and muscle function in both trained and contralateral untrained limbs in recreationally trained women. We hypothesize that supramaximal AEL will yield greater improvements in both limbs, providing insights into optimal eccentric loading strategies for women.

## 2. Materials and Methods

### 2.1. General Design

Female sports science students (*n* = 30) volunteered for the study and were randomly assigned to one of two training groups or a non-intervention control group. Participants in the training groups completed 20 sessions (4 sets × 8 repetitions) of the unilateral leg press with eccentric overload for the dominant leg over a 10-week period. Isotonic resistance was provided by a motorised exercise machine, with the concentric load for both groups set at 30% of the 1-RM and the eccentric phase at either 90% (submaximal load, SUB group) or 120% (supramaximal load, SUPRA group) of the concentric 1-RM, according to a previously described protocol [18]. Before and after training, unilateral leg press 1-RM, local muscle endurance, unilateral muscle strength at different percentages of 1-RM, unilateral vertical jump, thigh lean tissue mass (TTLM), and muscle soreness (assessed using a visual analogue scale) were assessed. All participants came to the laboratory for three appointments before the start of the training programme. On day 1, a dual-energy X-ray absorptiometry and vertical jump testing were performed. Twenty-four hours later, the leg press 1-RM tests was performed. Forty-eight hours later, mechanical power and muscle endurance tests in the same leg press exercise were performed. One week after training, these protocols were repeated in the same order and at the same time. On each day that participants attended the laboratory, a warm-up consisting of 5 min cycling, 25 reps of high knee, 25 reps of butt kicks, and two sets of 10 squat repetitions with their own body weight was performed.

### 2.2. Participants

In total, 34 sports science undergraduate students volunteered for the study (20.4 ± 2.0 years, 60.2 ± 7.1 kg, 165.1 ± 5.6 cm, and 32.1 ± 4.9 N/kg in the leg press exercise). Participants were moderately active and healthy, engaging in 6–8 h of recreational physical activity per week. They had a history of regular lower limb strength training for at least 1 year and no muscle, joint, or bone injuries in the last 6 months. They were informed of the purposes and risks involved in the study before providing their informed written consent to participate. Participants were instructed not to change their exercise habits and to refrain from performing additional resistance exercises during the experimental phase of the study. The Ethics Committee of the University of León approved the study protocols (ETICA-ULE-009-2018b). Four participants dropped out of the study: two due to musculoskeletal injuries unrelated to the study protocol and two due to scheduling conflicts. The remaining participants (*n* = 20) completed all study procedures as planned, including pre- and post-testing sessions, familiarization sessions, and the 20 training sessions. No a priori power analysis was conducted; however, the sample size is comparable to previous studies applying unilateral eccentric overload interventions.

### 2.3. Training Protocol

All experimental participants (SUB [*n* = 10] and SUPRA [*n* = 10]) completed 10 weeks (20 sessions) of an eccentrically accentuated unilateral leg press training programme using an electric-motor device (Exentrix, SmartCoach™, Stockholm, Sweden) according to a previously used design [18]. This device, controlled by a custom-designed power driver, precisely regulates both speed and torque, enabling accurate load prescription for each contraction [9]. Mean and peak force, velocity and power were measured during each concentric and eccentric contraction. Additionally, the time duration of each concentric and eccentric contraction was registered. Participants received real-time visual feedback and verbal encouragement during every repetition to ensure they maintained maximal concentric peak power output throughout the intervention. Participants in the SUB and SUPRA groups trained twice a week with a rest of at least 48 h between training sessions. After a standardised warm-up on a cycloergometer, participants performed 4 sets of 8 repetitions (including both concentric and eccentric muscle contractions) of unilateral (dominant leg) leg press exercise in a custom-built horizontal leg press machine [18]. The training device was configured in isotonic mode (i.e., constant load during the exercise) using the device software (Exentrix PC Interface—V2.4, SmartCoach™). Two different eccentric loading intensities were used: 90% of the concentric 1-RM (SUB group) and 120% of the concentric 1-RM (SUPRA group) [18]. The load in the concentric phase was 30% of 1-RM for both groups [25,29]. The transition time between eccentric and concentric actions was the minimum allowed by the system (0.5 s). Participants were instructed to exert maximal effort over the full range of motion (ROM), which ranged from 90° knee flexion to near-full extension (0° knee flexion). Participants in both experimental groups were instructed to perform the concentric phase as fast as possible and the eccentric phase in a controlled manner as slowly as possible, avoiding any pauses. Before each session, the ROM was set for each participant using a goniometer. Participants were blinded to the loading condition and were instructed not to use the untrained leg to generate force but to keep it fully extended and supported on a slider to minimise resistance during the exercise. Control group participants underwent only pre- and post-intervention assessments. Furthermore, all participants were explicitly requested to refrain from engaging in any additional strength training outside the laboratory for the duration of the study. The training load for both the SUB and SUPRA groups was maintained constant throughout the 8-week intervention, with no progressive overload applied. This approach allowed for a focused examination of the specific eccentric loading strategies.

### 2.4. Procedures

#### 2.4.1. Lean Tissue Mass

Dual-energy X-ray absorptiometry (DXA) scans were performed ~1 week before the first and ~1 week after the last exercise session at the same time of day using a Lunar Prodigy^®^ whole-body scanner (GE Medical Systems, Madison, WI, USA), following standardized protocols [30,31,32]. Participants reported in a fasting state, overnight sober, with an emptied bladder, and were asked to maintain consistent sleep and diet schedules before each scan [28]. The lean mass of each thigh was estimated according to previously validated procedures [9], with regions of interest manually defined for the whole thigh and subdivided into proximal, medial, and distal segments. Analyses were performed using Encore^®^ software (Lunar Corp., Madison, WI, USA), with a repositioning error of 1.2% determined from repeated scans.

#### 2.4.2. Unilateral Vertical Jump Performance

Following the DXA scan, vertical jumping performance was determined by unilateral countermovement jump (CMJ) and unilateral drop jump (DJ) tests on a contact platform (Globus Ergotester^®^, Globus, Codogne, Italy) [22]. Each test was performed separately with the dominant and non-dominant leg in a randomised order that was also maintained in the post-test evaluation. In the CMJ, participants began standing and were instructed to descend quickly and jump as high as possible with their hands on their hips. In the DJ, participants descended from a 30 cm high box and immediately jumped as fast as possible after landing. All jumps began with participants standing. The order of the tested legs was randomized for each participant, and this order was replicated during the post-test assessments. The jump height was measured with an accuracy of 0.1 cm and showed high reliability (ICC = 0.98, 95% CI: 0.95–0.99). For each test and leg, three trials were performed with a recovery time of 30 s between trials, and the best result was used for data analysis.

#### 2.4.3. Unilateral Maximal Dynamic Force

Twenty-four hours later, the unilateral one-repetition maximum (1-RM) load was determined on a 45° inclined leg press machine (Gerva-Sport, Madrid, Spain). Each participant performed the 1-RM test from full knee extension (0°) to 90° flexion and then to full extension with a load equivalent to approximately 3-RM. The load was increased by 10 kg if the participant succeeded or decreased by 5 kg if they failed. The test ended when each participant failed to overcome a given load in two consecutive trials. Both legs were tested in a randomized order, with a 2 min rest period between legs, and this order was replicated in the post-test. The unilateral 1-RM load was achieved between 3 and 6 trials, and trials were interrupted by a 2 min recovery period (participants were allowed to stand up during recovery periods). Participants adopted a testing position, with the knee of the unloaded leg flexed and the foot placed on the floor for support. The previously described warm-up was complemented with one set at ~8 RM load. The partial reliability (intraclass correlation coefficient, ICC) was 0.98 (95% CI: 0.93–0.99).

#### 2.4.4. Unilateral Maximal Dynamic Mechanical Power

Forty-eight hours after the 1-RM test, each participant completed 3 sets of three unilateral repetitions from full knee extension (lowered with control) to 90° flexion and then to full extension (0°) in the leg press, as described above, with a 2 min recovery between sets. To avoid the stretch-shortening cycle, each repetition began from a static position (the load was individually fixed with a safety belt at the exact point of 90° knee flexion). Sets were performed at 40, 60, and 80% of 1-RM, and the order of sets was individually randomised before the test and repeated in the post-exercise tests. Participants were asked to perform the concentric phase of each repetition as quickly as possible. The described protocol was performed in a randomised order for both legs, with a 2 min rest period, and this order was replicated during the post-test. Mean concentric mechanical power data for each repetition were recorded at 1000 Hz using an encoder (T-FORCE Dynamic Measurement System, Ergotech Consulting S.L., Murcia, Spain) and associated software (T-Force v. 2.28). The best repetition at each load was used for data analysis. Partial reliability (ICC) for unilateral concentric peak power was high at all loads (40%: 0.91 [95% CI: 0.81–0.96]; 60%: 0.93 [95% CI: 0.86–0.97]; 80%: 0.90 [95% CI: 0.80–0.96]).

#### 2.4.5. Unilateral Leg Press Muscle Endurance

After completing the mechanical power assessments for both legs, participants performed a 45°-inclined unilateral leg press repetition-to-failure (XRM) test at 60% of their individual 1-RM for the tested leg. The order of leg testing was randomised and maintained at post-test assessment. Accordingly, the same relative load was used before and after the training period, but a different absolute load was used. Participants were instructed to perform as many repetitions as possible while maintaining the cadence of 40 beats per minute set by the Google metronome. All repetitions performed between 90° knee flexion and full extension (0° knee flexion) were considered valid. The test was finished if the participant was unable to achieve full knee extension for two consecutive repetitions, and only successful repetitions were included in the analysis. The number of repetitions and the distance travelled during each repetition were measured using the previously described measurement device (T-FORCE Dynamic Measurement System, Ergotech Consulting S.L., Murcia, Spain) and its associated software (T-Force v. 2.28). No recovery periods were allowed between repetitions. Technical performance was supervised by a dedicated researcher, and participants received strong verbal encouragement. Only one maximal trial was performed, and the absolute number of repetitions was recorded.

#### 2.4.6. Muscle Soreness

Additionally, immediately after, and 2, 24, and 48 h after training sessions 1, 10, and 20, perceived muscle soreness was assessed using a 0–10 visual analogue scale (VAS) with a 100 mm horizontal line with “no pain” on one end (0 mm, 0 points) and “extremely painful” on the other (100 mm, 10 points). Participants were asked to mark the perceived pain level during a functional activity (i.e., standing to sitting position) on the VAS.

#### 2.4.7. Statistical Analyses

All statistical analysis was performed using the Jamovi software package (The Jamovi Project, v.1.6.23.0; downloadable at https://www.jamovi.org, accessed on 22 january 2026). Normality was checked by the Shapiro–Wilk normality test. Then, a repeated-measures linear mixed model, fitted with a restricted maximum likelihood method and unstructured covariance, with Tukey post hoc adjustment, was used to compare outcomes between time (pre vs. post), leg (trained vs. non-trained), and training protocol (SUB vs. SUPRA vs. control). The fixed factors were time, leg, and protocol, with participants included as random effects. Model fit was assessed by Akaike’s Information Criterion (AIC) and Bayesian Information Criterion (BIC), and significant group × time × leg interactions were examined to better interpret cross-education effects. The effect size (ES) was calculated for interactions between groups using Hedges’s guidelines. Threshold values for ES were >0.2 (small), >0.6 (large), and >2.0 (very large) [33,34]. The mean, standard error (SE), and t value were reported for all statistical analyses. In addition, Pearson’s r was used to examine correlations between TL and NTL for the percent changes in each dependent variable from pre- to post-training for the participants in the training groups. For all statistical analyses, a significance level was set at *p* < 0.05. Results are reported as mean ± SD.

## 3. Results

### 3.1. Training

The average peak power for both concentric and eccentric contractions increased (*p* < 0.01) from the first to the last training session, but the increases were similar between SUB (concentric: 32%, 420.1 ± 126.9–553.8 ± 75.3 W; eccentric: 20%, 622.3 ± 136.5–741.7 ± 39.9 W) and SUPRA groups (concentric: 42%, 427.9 ± 90.7–607.3 ± 76.8 W, 36%; eccentric: 22%, 732.3 ± 238.5–893.7 ± 126.1 W). Similarly, the mean concentric and eccentric velocity increased (*p* < 0.01) from the first to the last training session, but the increases were similar between SUB (concentric: 25%, 1.96 ± 0.2–2.44 ± 0.5 m/s; eccentric: 45%, 0.73 ± 0.2–1.06 ± 0.2 m/s) and SUPRA groups (concentric: 29%, 2.12 ± 0.2–2.73 ± 0.2 m/s; eccentric: 25%, 0.84 ± 0.2–1.05 ± 0.3 m/s). All training sessions involved eccentric overload. The mean eccentric overload, expressed as the percentage of eccentric average peak power relative to the concentric average peak power, ranged from 50.9 ± 21.2% to 36.3 ± 22.3% in SUB and from 94.7 ± 43.5% to 47.7 ± 17.6% in SUPRA, from session 1 to session 20, respectively. The analysis of the time under tension revealed that both groups maintained similar durations in the concentric and eccentric phases across the intervention. In the SUB group, the concentric phase lasted 0.33 ± 0.0 s at PRE and 0.28 ± 0.0 s at POST, whereas the eccentric phase lasted 1.61 ± 0.5 s at PRE and 1.86 ± 0.4 s at POST. Similarly, in the SUPRA group, the concentric phase lasted 0.34 ± 0.1 s at PRE and 0.29 ± 0.0 s at POST, while the eccentric phase lasted 1.32 ± 0.3 s at PRE and 1.64 ± 0.4 s at POST. No significant differences were found either between groups or across time points for either phase. In addition, no significant differences were observed between groups before intervention for any outcome.

### 3.2. Lean Tissue Mass

**Figure 1:**
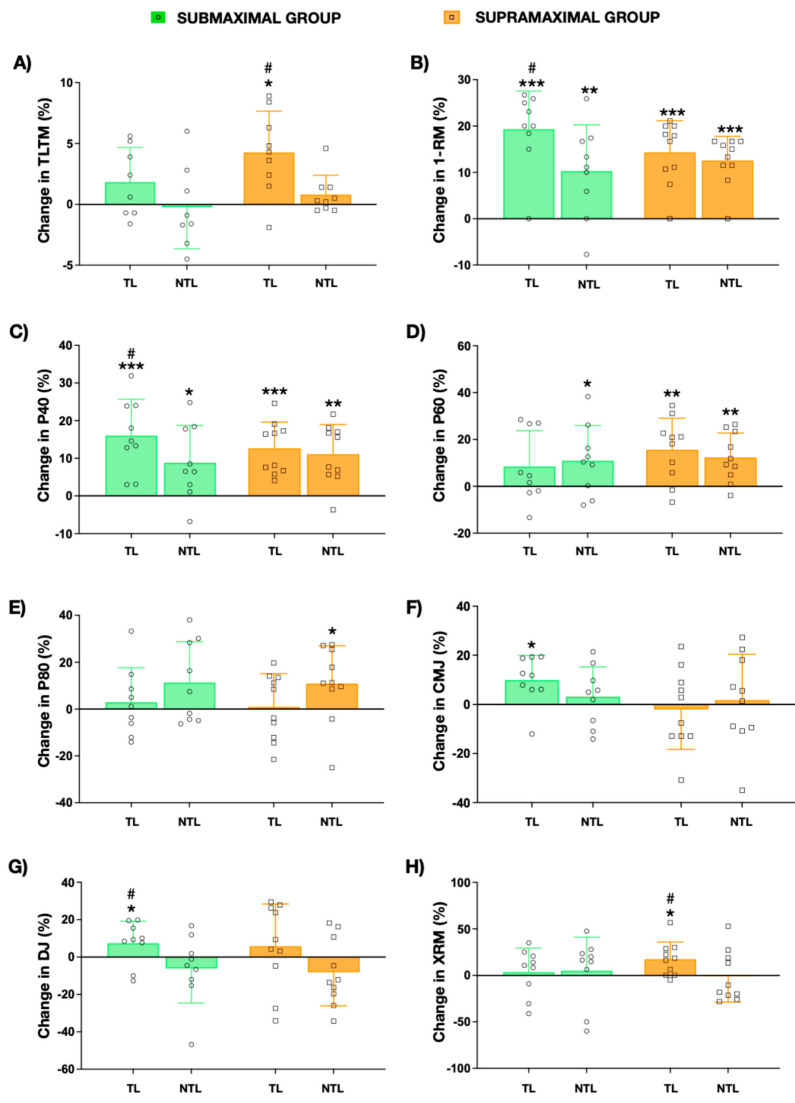
Relative changes from the baseline values (mean ± SD) in thigh lean mass (TTLM, (**A**)), one-repetition maximal of one leg press (1-RM, (**B**)), concentric mean power at 40% (P40, (**C**)), 60% (P60, (**D**)), and 80% of 1-RM (P80, (**E**)), countermovement jump height (CMJ, (**F**)), drop jump height (DJ, (**G**)) and for muscle endurance at 60% of the 1-RM in the leg press exercise (XRM, (**H**)) for the trained leg (TL) and non-trained leg (NTL) in the submaximal (green) and supramaximal (orange) groups. * A significant (*p* < 0.05), ** *p* < 0.01, *** *p* < 0.001. change from the baseline. # A significant (*p* < 0.05) difference from the trained leg.

A statistically significant increase in TTLM was only observed in the TL of the SUPRA group (4.55%, *p* = 0.005; 95% CI: 76.99–309.23; ES = 0.39; see Figure 1A and Table 1), whereas no significant changes were found in NTL. The SUB group exhibited no significant differences in TTLM in either leg. In addition, when comparing the lean tissue mass changes between TL and NTL, significant differences (*p* = 0.008) were observed at post-training in the SUPRA group.

### 3.3. Muscle Strength

Regarding muscular 1-RM, both groups demonstrated significant increases after training in both the TL (SUPRA: 16.29%, *p* < 0.001; 95% CI: 11.87–24.13, ES = 0.77; and SUB: 25.00%, *p* < 0.001; 95% CI: 18.16–40.72, ES = 0.97) and the NTL (SUPRA: 14.22%, *p* < 0.001; 95% CI: 10.23–19.77; ES = 0.75; and SUB: 11.20%, *p* = 0.019; 95% CI: 2.77–22.78; ES = 0.51) as shown in Figure 1B and Table 1. However, significant differences (*p* = 0.015) were observed between TL and NTL in the SUB group at post-training. A significant positive correlation was observed between 1-RM magnitude of change in the NTL and in the TL in the SUPRA group (R = 0.933, R^2^ = 0.871, *p* < 0.001, Supplementary File S1). When the data from both experimental groups were pooled, a significant correlation was observed between changes in 1-RM in the NTL and TL (R = 0.563, R^2^ = 0.317, *p* = 0.012; Supplementary File S1).

### 3.4. Mechanical Power

At low intensity, both groups showed significant improvements after training in P40 in both the TL (SUPRA: 15.02%, *p* < 0.001, 95% CI: 11.87–24.13; ES = 0.67; and SUB: 18.71%, *p* < 0.001, 95% CI: 27.38–67.73; ES = 0.75) and the NTL (SUPRA: 12.73%, *p* = 0.003; 10.23–19.77; ES = 0.66; SUB: 9.60%, *p* = 0.022; 95% CI: 4.37–43.19; ES = 0.53; see Figure 1C and Table 1). In addition, significant differences (*p* = 0.002) were observed between TL and NTL in the SUB group when the magnitude of change was compared. Furthermore, a positive correlation was observed in the SUB group between changes in NTL and changes in TL (R = 0.881, R^2^ = 0.777, *p* = 0.002, Supplementary File S1). Although no significant correlation between leg changes was observed for SUPRA, the pooled data from both groups showed a significant correlation between changes in NTL and TL at P40 (R = 0.493, R^2^ = 0.243, *p* = 0.031; Supplementary File S1). At medium intensity, significant increases were only observed for the SUPRA group (TL: 18.46%, *p* = 0.006; 95% CI: 17.91–78.08; ES = 0.75; NTL: 12.73%, *p* = 0.006; 95% CI: 14.31–62.29; ES = 0.74; see Figure 1D and Table 1). Although no significant differences were observed in the TL within the SUB group, a significant increase in P60 was found in the NTL (14.73%, *p* = 0.043; 95% CI: 1.27–39.23; ES = 0.74). For P80, only the SUPRA group showed significant changes in the NTL (15.51%, *p* = 0.040; 95% CI: 2.12–35.38; ES = 0.62). No significant differences between the SUPRA and SUB groups were observed for any load (Table 1), nor were any significant differences found between TL and NTL within either the SUPRA or SUB group (Figure 1C–E). There were no significant correlations between the magnitude of change in NTL and TL within the SUPRA group or at P60 and P80 within the SUB group.

### 3.5. Vertical Jump Performance

Significant differences were only found for the CMJ in the SUB group for TL (11.35%, *p* = 0.022; 95% CI: 0.30–3.01; ES = 0.65; see Figure 1F and Table 1). No significant differences were observed between TL and NTL for either the SUPRA or SUB group. Similarly, the SUB group showed a significant increase in the DJ in the TL (11.04%, *p* = 0.045; 95% CI: 0.04–3.18, ES = 0.45; see Figure 1G and Table 1). When comparing TL and NTL, significant differences were observed in the SUB group (*p* = 0.032), while no significant differences were observed for the SUPRA group. No significant correlations between legs were observed for any vertical jump test.

### 3.6. Local Muscular Endurance

The SUPRA group showed a significant increase in XRM in the TL (17.9%, *p* = 0.022, 95% CI: 0.842–8.358; ES = 0.31; see Figure 1H and Table 1). When comparing TL and NTL, significant differences (*p* = 0.006; ES = 1.13) were observed between legs in the SUPRA group. Moreover, a significant correlation was observed between NTL and TL changes in XRM for SUPRA (R = 0.817, R^2^ = 0.668, *p* = 0.004; Supplementary File S1). No significant changes were observed in the SUB group in XRM.

### 3.7. Muscle Soreness

Finally, regarding muscle soreness, no significant differences were observed between groups at any time for any training session (please see Supplementary Table S1).

## 4. Discussion

The 10-week AEL intervention (20 sessions) did not reveal distinct adaptive patterns between the TL and NTL across different loading intensities in recreationally trained women. Submaximal loading induced greater 1-RM improvements in TL compared to NTL, while supramaximal AEL produced comparable enhancements in both limbs. Mechanical power output adaptations showed protocol-specific variations: submaximal AEL elicited significant changes only with low loads, whereas supramaximal AEL generated significant improvements at low and medium intensities in both limbs. In addition, unilateral CMJ and unilateral DJ performance only increased after submaximal loading in TL. TTLM increases in TL only after supramaximal loading, although without significant improvements in NTL. The correlation between TL and NTL was moderate for P60, 1-RM, and MVIC after submaximal AEL. However, the correlation between the two legs was strong for P40 after submaximal AEL and for 1-RM and XRM after supramaximal AEL. These results indicate a strong cross-education effect of sub- and supramaximal AEL with no difference between them in recreationally trained women.

RT has long been considered the best training method for increasing lean mass, strength, power, and muscular endurance. Recently, accentuated eccentric loading (AEL) has emerged as a promising strategy to enhance RT outcomes. This approach is based on the fact that eccentric contractions require lower motor unit recruitment and firing rates than concentric contractions at the same load, which allows for the use of heavier eccentric loads [35]. Research shows that eccentric overload not only increase force production in the following concentric muscle contraction but also promotes the recruitment of high-threshold motor units, which supports improved neuromuscular performance [5]. Additionally, muscle architecture plays a role, as fascicles operate closer to their optimal length and angle during these movements [36]. AEL also stimulates Type Ia afferents, triggering a stretch reflex, and allows elastic energy stored in muscle and connective tissue (partly due to titin) to be released during the concentric action [37], resulting in a significant anabolic response and improved explosive strength, as evidenced by enhanced jump performance and a faster and stronger muscle phenotype [16]. Collectively, these mechanisms contribute to neuromuscular adaptations that can result in greater gains in hypertrophy, strength, and power than other RT methods [7,38].

Our results showed that both submaximal and supramaximal loading increased muscle strength in both TL and NTL, with higher increases in TL compared to NTL after submaximal loading, with no difference between the loading patterns. Maroto-Izqierdo et al. [18] reported difference in 1-RM just for supramaximal but not for submaximal loading in men. The discrepancy between our findings and those of Maroto-Izquierdo et al. [18] may be attributed to differences in participant gender, as our study included females while theirs focused on males. Previous studies have suggested that strength gains may be more evenly distributed with submaximal loading in female participants in both the TL [27] and the NTL [39]. These results demonstrate a similar magnitude of cross-education between loading approaches, highlighting that the use of supramaximal loads is not necessary to enhance contralateral strength gains in recreationally trained women.

Although no previous studies have directly compared the effects of submaximal versus supramaximal AEL on 1-RM cross-education, existing research consistently demonstrates the positive impact of unilateral eccentric training on the lower limbs [19,20,39,40,41]. The cross-education effect is primarily explained by two neural mechanisms: cross-activation, where neural drive from the trained side ‘spills over’ to the untrained side, and bilateral access, where adaptations in the trained limb’s motor system are accessible to the opposite limb [40,42]. Additionally, changes in the excitability of ipsilateral neural pathways and non-decussated corticospinal projections may facilitate strength transfer, especially in the lower limbs [43]. These mechanisms likely coexist, resulting in subtle but significant strength gains in the untrained limb after unilateral resistance training [42,44]. Specifically, eccentric training may uniquely modulate corticospinal excitability and inhibition in the untrained limb to a greater extent than concentric training [45], potentially inducing a shift in motor unit recruitment and enhanced α-motoneuron excitability in contralateral limb muscles [46]. This effect may be accentuated by the higher speeds and loads inherent to AEL [23], suggesting that accentuated eccentric loading provides a unique stimulus, promoting greater neural adaptations compared with traditional loading [22]. However, further research is needed to explicitly test this hypothesis by comparing it to a non-eccentrically overloaded group.

Our results demonstrated that power output varied depending on intensity used. For P40, both the TL and NTL showed a significant increase in power under both submaximal and supramaximal loading. Notably, under submaximal loading, the increase in TL was significantly greater than in NTL. At P60, NTL increased in both groups, whereas TL did not in SUB. At P80, only SUPRA showed significant improvements in mechanical power, while submaximal loading induced no changes in either TL or NTL. These findings highlight a load-dependent pattern in the response of mechanical power. Additionally, since the exercise intensity in the concentric phase was set at 30% of 1-RM, based on the specificity principle, we observed increased power primarily in P40 but not in the higher loads [47]. Maroto-Izquierdo et al. [22] reported that six weeks of isoinertial single-leg squat training (twice weekly) using an electric-motor device resulted in increases in P40, P60, and P80 in both TL and NTL. Furthermore, the use of a flywheel device for the same exercise and training volume resulted in significant increases in both P60 and P80, with no notable difference between the two legs. In contrast, for P40, a significant improvement was observed only in TL. The discrepancies observed in P40 and P60 between our study and that of Maroto-Izquierdo et al. may underscore the importance of training with maximal or near maximal concentric loads to enhance mechanical power, irrespective of the magnitude of eccentric overload. Regarding vertical jump performance, changes in TL were similar to previous studies using submaximal accentuated eccentric loading [9,14,15,22]. We observed significant changes in unilateral CMJ and DJ in the TL only under the submaximal loading pattern. However, in contrast to the findings reported by Maroto-Izquierdo et al. [22], we did not observe effects in the NTL despite employing a high magnitude of eccentric overload. This suggests that, to enhance vertical jump performance in the NTL, not only is a high eccentric overload required but also a maximal concentric contraction that optimizes the stretch-shortening cycle and a rapid eccentric velocity—key differences from the aforementioned study. Future research should explore not only the effect of eccentric load magnitude but also the impact of different eccentric velocities with submaximal loads on contralateral limb adaptations in vertical jump performance.

In contrast to muscle strength, XRM only changed in the TL after supramaximal loading. However, the review by Song et al. [48] emphasises that the current literature does not provide sufficient evidence to draw firm conclusions regarding the existence of cross-education for muscular endurance. Furthermore, none of the studies included in this review utilized eccentric overload. This variability may be attributed to differences in study design, participant characteristics, training protocols, and the methods used to assess endurance. Additionally, the neural mechanisms that underpin cross-education of strength may not translate as effectively to muscular endurance, which is also influenced by local muscular and metabolic adaptations that are less likely to occur in the untrained limb. Therefore, our results add to the growing body of evidence suggesting that, although cross-education is a robust phenomenon for strength, its effects on muscular endurance are less consistent and may require further investigation using standardized protocols and larger sample sizes.

Despite previous studies having correlated strength gains in the contralateral limb with increases in contralateral lean mass, these changes may be explained by systemic anabolic responses mediated by growth factors and myokines, low-level muscle activation for stabilization, alterations in muscle water content, and potential measurement limitations of DXA [22]. In this study, lean mass in the NTL did not increase significantly under any of the conditions investigated. A significant increase in lean mass was only observed in TL in the SUPRA group. These results are consistent with those reported by Fernandez-Gonzalo et al. [28] in women performing unilateral leg press training with eccentric overload using flywheel technology. Therefore, the results of this study are consistent with previous research, as the CE effect is mainly mediated by neural adaption [40].

The most important finding of this study is the lack of a significant difference between sub- and supramaximal CE load in all variables. This is in line with emerging findings on neurally driven adaptations during eccentric training. While earlier studies suggested that higher loads improve cross-education (e.g., 80% vs. 40% 1-RM in elbow flexion training) [49], more recent work points to eccentric-specific mechanisms that reduce load dependence [43,50,51]. For example, Walker et al. [10] reported comparable MVIC gains in both limbs regardless of eccentric load magnitude, attributed to enhanced motor unit synchronization. This highlighted that submaximal and supramaximal accentuated eccentric loading both reduce cortical inhibition and increase corticospinal drive to untrained limbs with significant difference among them. In addition, eccentric actions inherently recruit high-threshold motor units at lower perceived efforts, potentially saturating neural adaptations even at submaximal loads [51]. Hortobágyi et al. [52] observed similar cross-education effects between 80% and 120% 1-RM eccentric protocols, noting diminished returns beyond 90% 1-RM due to plateauing neural drive [18].

### Limitations

Several limitations of this study should be noted. First, the intervention period was limited to 10 weeks of training twice per week, which may not have been sufficient to observe the full extent of neuromuscular and morphological adaptations, particularly in the untrained limb. Future studies should consider longer training durations to better capture the time course of these adaptations. Secondly, the sample consisted exclusively of young, physically active women with a relatively homogeneous profile in terms of age, training experience, and baseline strength. The generalizability of these results to other population groups such as men, older adults, or individuals with different training backgrounds therefore remains uncertain. Third, the study did not include a traditional concentric–eccentric or purely eccentric control group, which limits the ability to directly compare the effects of accentuated eccentric loading with other modalities of resistance training. In addition, the use of DXA to assess changes in lean mass, while widely accepted, has inherent limitations, including an inability to differentiate between specific muscle groups and sensitivity to variations in hydration status. Although efforts have been made to standardize conditions prior to measurement, residual bias due to changes in muscle water content or other factors cannot be completely ruled out. Fourth, a limitation of the present study is that hormonal status and menstrual cycle phases were not controlled, which may influence neuromuscular adaptations and deserve further investigation. Finally, the relatively small sample size may have limited the statistical power to detect subtle differences between groups or conditions. Future research should address these limitations by including larger and more diverse samples, longer intervention periods, and additional control groups to further explore optimal eccentric loading strategies to improve lateral formation and neuromuscular adaptations in women.

## 5. Conclusions

In summary, this study shows that both submaximal and supramaximal AEL produces significant cross-formational effects on strength, power and muscle mass, with no significant difference between exercise intensities in recreationally trained women; however, these results should be interpreted with caution given the small sample size. While strength and power gains were consistently observed in both the trained and untrained limbs, hypertrophic adaptations in the untrained limbs were only significant at supramaximal loading, indicating a potential for limited cross-training of muscle mass under certain conditions. The lack of a clear load-dependent effect on cross-education, particularly for neural outcomes, underpins the growing consensus that accentuated eccentric training induces robust neural adaptations even at submaximal intensities. However, the effects on muscular endurance remain inconsistent, highlighting the need for further research using standardised protocols to clarify the mechanisms and optimise the application of AEL. These findings provide preliminary evidence that unilateral AEL can enhance neuromuscular performance and cross-education effects in women, and importantly, that such adaptations can be achieved without the need for supramaximal loads, underscoring the practical applicability of submaximal protocols in both rehabilitation and athletic training contexts.

## Figures and Tables

**Table 1:** Changes (mean ± SD) in unilateral total thigh lean mass (TTLM), one-repetition maximal strength (1-RM), muscle power at different loads of the 1-RM (40%, 60%, and 80% 1-RM loads, P40, P60, and P80, respectively), countermovement (CMJ) and drop jump (DJ) heights, and muscle endurance at 60% of the 1-RM for the unilateral leg press exercise for the trained (TL) and non-trained legs (NTL) for each group (SUB, SUPRA, and CONTROL) before (pre) and after training (post), *p* value for the comparison between pre- and post-training values by Tukey test, and the effect size (ES) for the changes are shown for each leg.

	Trained Leg				Non-Trained Leg			
	Pre	Post	P	ES	Pre	Post	P	ES
SUB group (*n* = 10)								
TTLM (g)	4294.9 ± 456.1	4373.1 ± 420.2	0.111	0.18	3705.3 ± 379.6	4208.1 ± 432.7	0.352	0.49
1-RM (kg)	117.8 ± 25.0	147.2 ± 34.1	<0.001	0.97	113.9 ± 26.9	126.7 ± 24.4	0.019	0.51
P40 (W)	254.2 ± 64.1	301.8 ± 63.7	<0.001	0.75	247.6 ± 46.2	271.3 ± 39.8	0.022	0.53
P60 (W)	289.2 ± 73.4	315.3 ± 55.8	0.125	0.35	274.7 ± 52.1	310.9 ± 49.1	0.043	0.58
P80 (W)	268.4 ± 51.5	277.1 ± 36.8	0.559	0.20	253.0 ± 52.4	288.2 ± 41.4	0.076	0.75
CMJ (cm)	14.6 ± 2.6	16.3 ± 2.6	0.022	0.65	15.0 ± 2.5	15.6 ± 2.0	0.404	0.27
DJ (cm)	14.6 ± 2.5	16.2 ± 4.3	0.045	0.45	15.2 ± 2.7	14.5 ± 2.3	0.345	0.28
XRM (n)	28.7 ± 8.5	31.6 ± 11.9	0.256	0.28	26.4 ± 5.3	30.9 ± 10.6	0.170	0.59
SUPRA group (*n* = 10)								
TTLM (g)	4245.1 ± 472.3	4438.2 ± 514.9	0.005	0.39	4180.1 ± 488.1	4212.9 ± 488.9	0.205	0.07
1-RM (kg)	110.5 ± 23.6	128.5 ± 24.3	<0.001	0.77	105.5 ± 19.5	120.5 ± 20.9	<0.001	0.75
P40 (W)	242.3 ± 48.2	278.7 ± 58.4	<0.001	0.67	228.6 ± 41.8	257.7 ± 46.3	0.003	0.66
P60 (W)	260.0 ± 68.5	308.0 ± 59.2	0.006	0.75	258.0 ± 47.0	296.3 ± 56.4	0.006	0.74
P80 (W)	264.2 ± 64.3	268.4 ± 58.1	0.729	0.07	245.0 ± 43.2	283.3 ± 74.6	0.040	0.62
CMJ (cm)	15.2 ± 1.6	15.1 ± 1.6	0.870	0.06	14.6 ± 1.3	15.2 ± 2.4	0.509	0.31
DJ (cm)	14.5 ± 2.8	15.8 ± 3.2	0.246	0.43	14.9 ± 3.6	13.8 ± 2.3	0.219	0.36
XRM (n)	25.7 ± 14.2	30.3 ± 12.1	0.022	0.31	31.0 ± 18.5	30.8 ± 14.4	0.947	0.02
CONTROL group (*n* = 10)								
TTLM (g)	3838.1 ± 165.3	3817.2 ± 162.9	0.995	0.13	3845.1 ± 280.1	3816.9 ± 160.9	0.999	0.13
1-RM (kg)	143.0 ± 18.8	109.1 ± 18.3	0.454	1.74	109.5 ± 7.8	103.2 ± 6.9	0.599	0.80
P40 (W)	192.3 ± 20.2	214.1 ± 19.9	0.494	1.10	198.2 ± 19.4	219.0 ± 16.1	0.563	1.20
P60 (W)	237.1 ± 21.5	248.4 ± 16.0	0.950	0.59	214.1 ± 19.0	226.3 ± 17.4	0.954	0.67
P80 (W)	203.2 ± 17.3	218.4 ± 14.8	0.824	0.94	204.0 ± 15.5	219.3 ± 19.6	0.440	0.85
CMJ (cm)	13.7 ± 0.8	14.4 ± 0.9	0.936	0.82	13.4 ± 0.8	14.0 ± 0.8	0.958	0.75
DJ (cm)	13.7 ± 0.9	14.2 ± 1.3	0.995	0.45	13.3 ± 1.2	13.8 ± 0.9	0.991	0.47
XRM (n)	21.7 ± 3.5	23.9 ± 3.4	0.953	0.58	23.0 ± 3.8	24.0 ± 3.6	0.999	0.27

Abbreviations: 1-RM, one-repetition maximum strength; TTLM, total thigh lean mass; CMJ, countermovement jump height; DJ, drop jump height; P40, concentric mean power at 40% of 1-RM; P60, concentric mean power at 60% of 1-RM; P80, concentric mean power at 80% of 1-RM; and XRM, muscle endurance at 60% of the 1-RM in the leg press exercise.

## Data Availability

Data will be made available upon reasonable request.

## Supplementary Materials

The following supporting information can be downloaded at https://www.mdpi.com/article/10.3390/jfmk11010063/s1, Supplementary File S1: Associations between changes in muscle function in the trained leg (TL) and the non-trained leg (NTL) after 8 weeks of unilateral eccentric overload resistance training. Scatter plots depict correlations in submaximal (SUB, green) and supramaximal (SUPRA, orange) groups, as well as the pooled sample (blue). Data are shown for concentric peak power at 40% 1-RM (P40), peak power at 60% 1-RM (P60), one-repetition maximum strength (1-RM), and maximal number of repetitions (XRM). Coefficients of determination (R^2^) are displayed for each regression line. Positive correlations indicate that greater improvements in the trained leg were associated with greater cross-education effects in the contralateral non-trained leg. Supplementary Table S1: Perceived muscle soreness (VAS_0–10_) mean ± SD values for both submaximal (SUB) and supramaximal groups (SUPRA) immediately after training (0 h) and after 2, 24, and 48 h post-training, *p* value for the comparison between SUB and SUPRA values, effect size (ES) and mean differences, and 95% CIs are shown for each session and measure.

## Abbreviations

The following abbreviations are used in this manuscript:

1-RM	One-repetition maximum
CE	Cross-education
CI	Confidence interval
CMJ	Countermovement jump
DJ	Drop jump
DXA	Dual energy X-ray absorptiometry
ES	Effect size
NTL	Non-trained leg
P40	Mean concentric mechanical power at 40% of 1-RM
P60	Mean concentric mechanical power at 60% of 1-RM
P80	Mean concentric mechanical power at 80% of 1-RM
ROI	Region of interest
ROM	Range of motion
RT	Resistance training
SE	Standard error
SUB	Submaximal load group
SUPRA	Supramaximal load group
TL	Trained leg
TTLM	Total thigh lean mass
VAS	Visual analogue scale
XRM	Repetition-to-failure test
