# Supplementary material for: Cross-Education Effects After Submaximal and Supramaximal Accentuated Eccentric Loading on Lean Mass and Function in Women
Source: J Funct Morphol Kinesiol. 2026 Jan 31;11(1):63. doi: 10.3390/jfmk11010063 (PMC12921896; doi:10.3390/jfmk11010063)
Supplement: Supplementary file 1 [file jfmk-11-00063-s001.zip › jfmk-4005074-Supplementary Table S1.pdf]

Supplementary Table S1. Perceived muscle soreness (VAS_{0-10}) mean ± SD values for both submaximal (SUB) and supramaximal groups (SUPRA) immediately after training (0h) and after 2-, 24- and 48-h post-training, *p* value for the comparison between SUB and SUPRA values, effect size (ES) and mean differences and 95% CI are shown for each session and measure.

	**SUB**	**SUPRA**	***p***	**ES**	**Mean (95% CI)**
**Session 1**					
$VAS_{(0-10)}$ 0 h	3.0 ± 2.0	3.3 ± 2.6	0.872	0.08	0.2 (-2.8 - 2.4)
$VAS_{(0-10)}$ 2 h	3.6 ± 1.8	2.6 ± 2.5	0.422	0.42	1.0 (-3.5 - 1.6)
$VAS_{(0-10)}$ 24 h	2.2 ± 1.8	2.3 ± 3.1	0.925	0.05	0.1 (-2.9 - 3.1)
$VAS_{(0-10)}$ 48 h	0.8 ± 1.2	0.5 ± 1.0	0.506	0.35	0.3 (-1.6 - 0.9)
**Session 10**					
VAS(0-10) 0 h	3.0 ± 2.4	2.4 ± 2.8	0.656	0.22	0.6 (-3.4 - 2.2)
VAS(0-10) 2 h	1.6 ± 2.0	2.1 ± 2.4	0.608	0.25	0.6 (-1.8 – 2.9)
VAS(0-10) 24 h	0.9 ± 1.4	1.6 ± 2.3	0.460	0.37	0.7 (-1.3 - 2.8)
VAS(0-10) 48 h	0.6 ± 1.3	1.0 ± 1.7	0.629	0.24	0.4 (-1.3 - 2.0)
**Session 20**					
VAS(0-10) 0 h	2.2 ± 1.7	1.7 ± 1.9	0.585	0.29	0.5 (-2.6 - 1.5)
VAS(0-10) 2 h	1.4 ± 1.9	1.3 ± 2.0	0.966	0.02	0.0 (-2.2 - 2.2)
VAS(0-10) 24 h	1.0 ± 1.8	1.1 ± 1.9	0.887	0.07	0.1 (-2.3 - 2.0)
VAS(0-10) 48 h	0.2 ± 0.2	0.8 ± 2.0	0.500	0.43	0.7 (-1.64 - 3.1)
