# Supplementary figures and images for: Cross-Education Effects After Submaximal and Supramaximal Accentuated Eccentric Loading on Lean Mass and Function in Women
Source: J Funct Morphol Kinesiol. 2026 Jan 31;11(1):63. doi: 10.3390/jfmk11010063 (PMC12921896; doi:10.3390/jfmk11010063)

..... SUBMAXIMAL GROUP    ..... SUPRAMAXIMAL GROUP    — POOLED GROUP

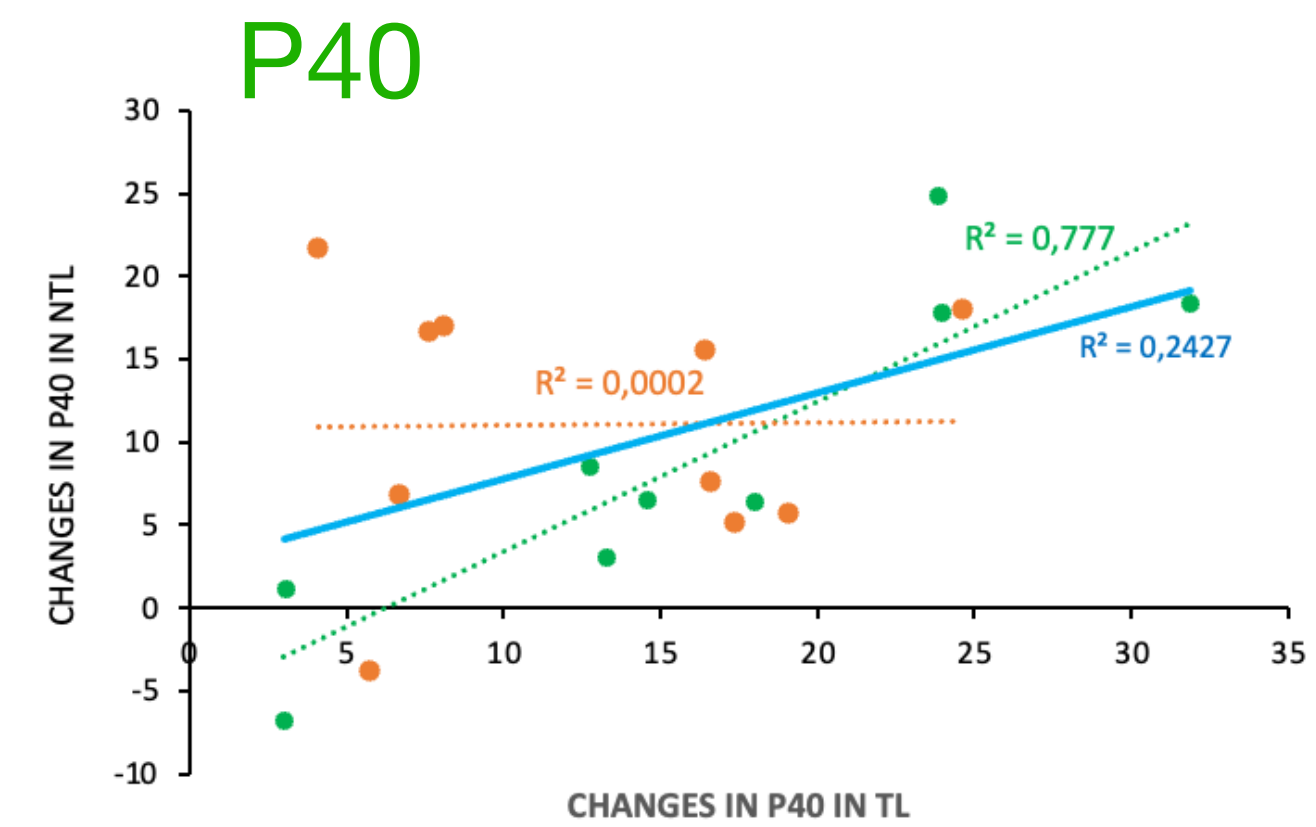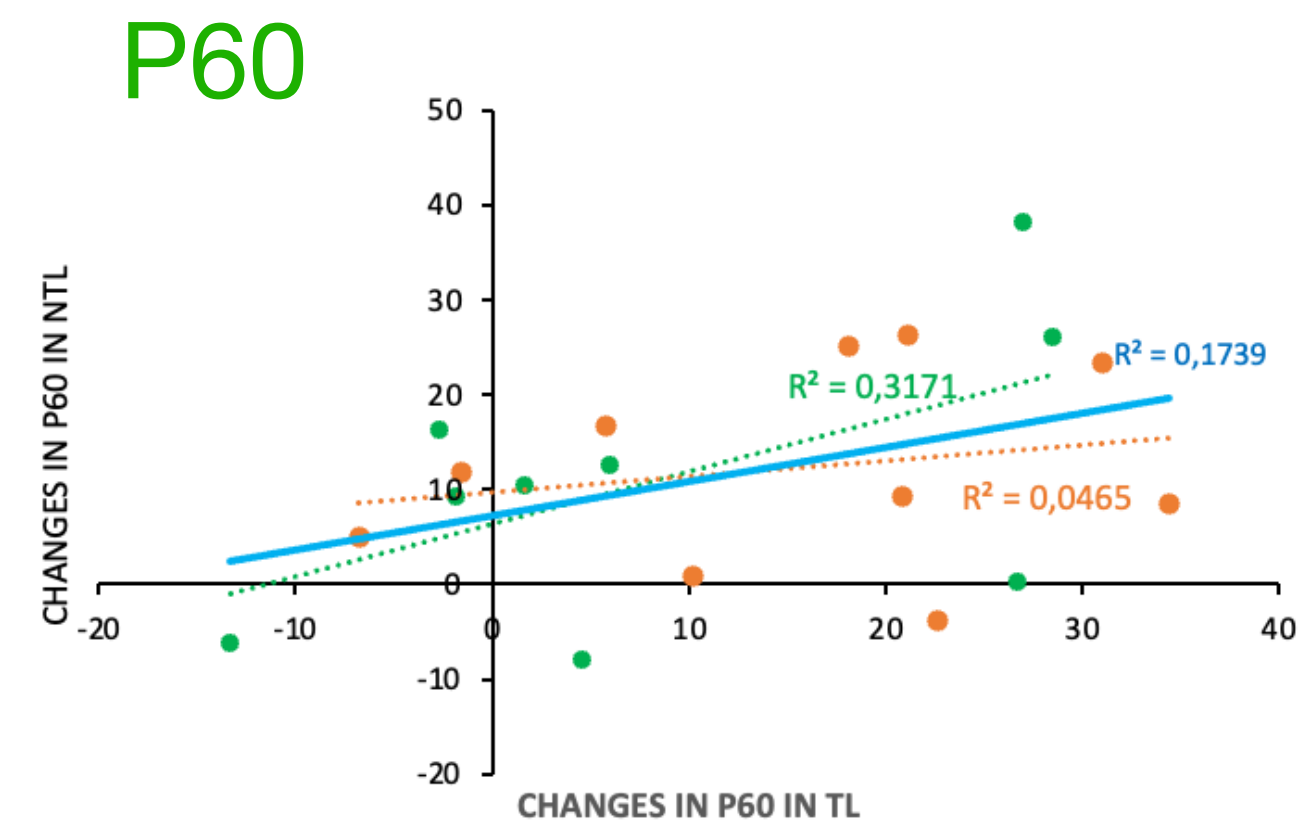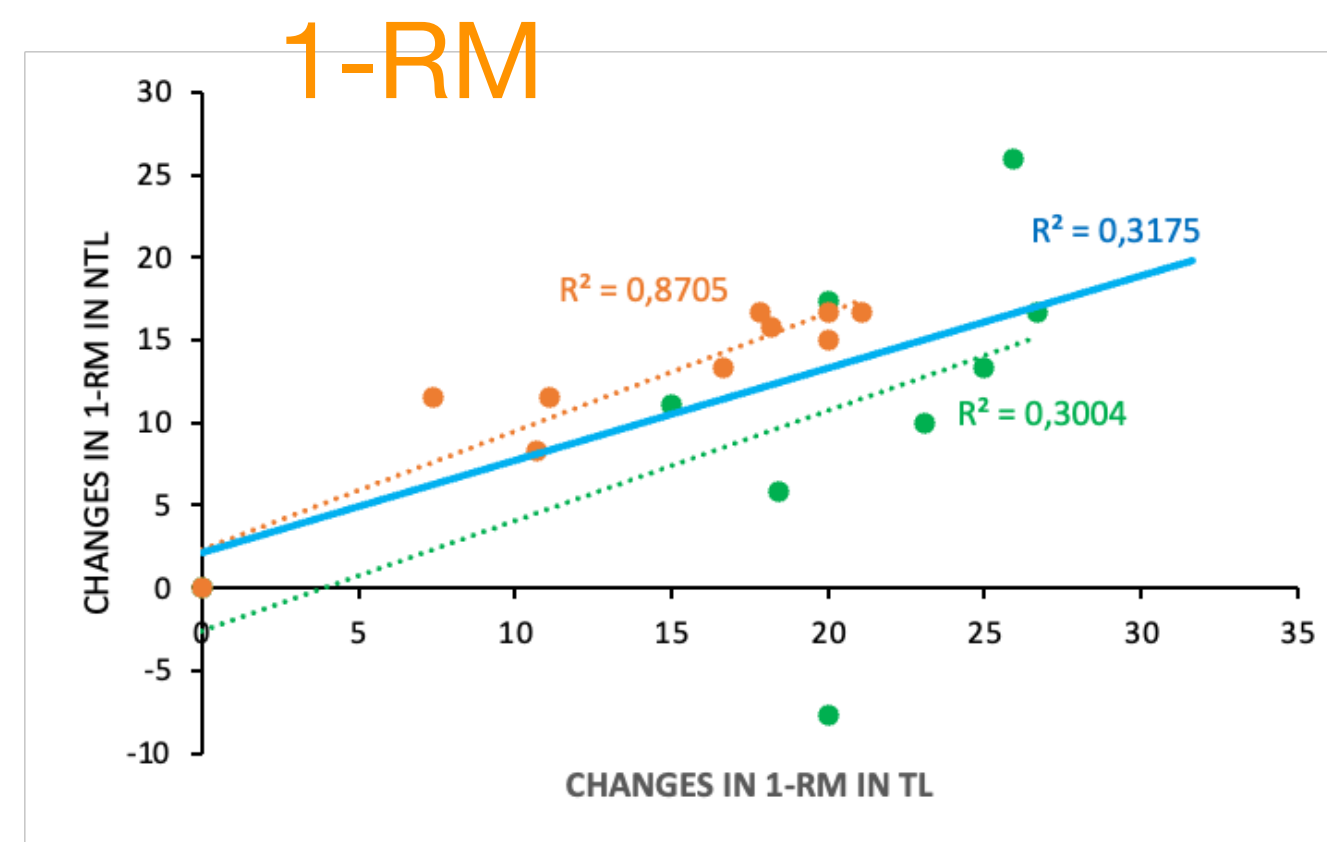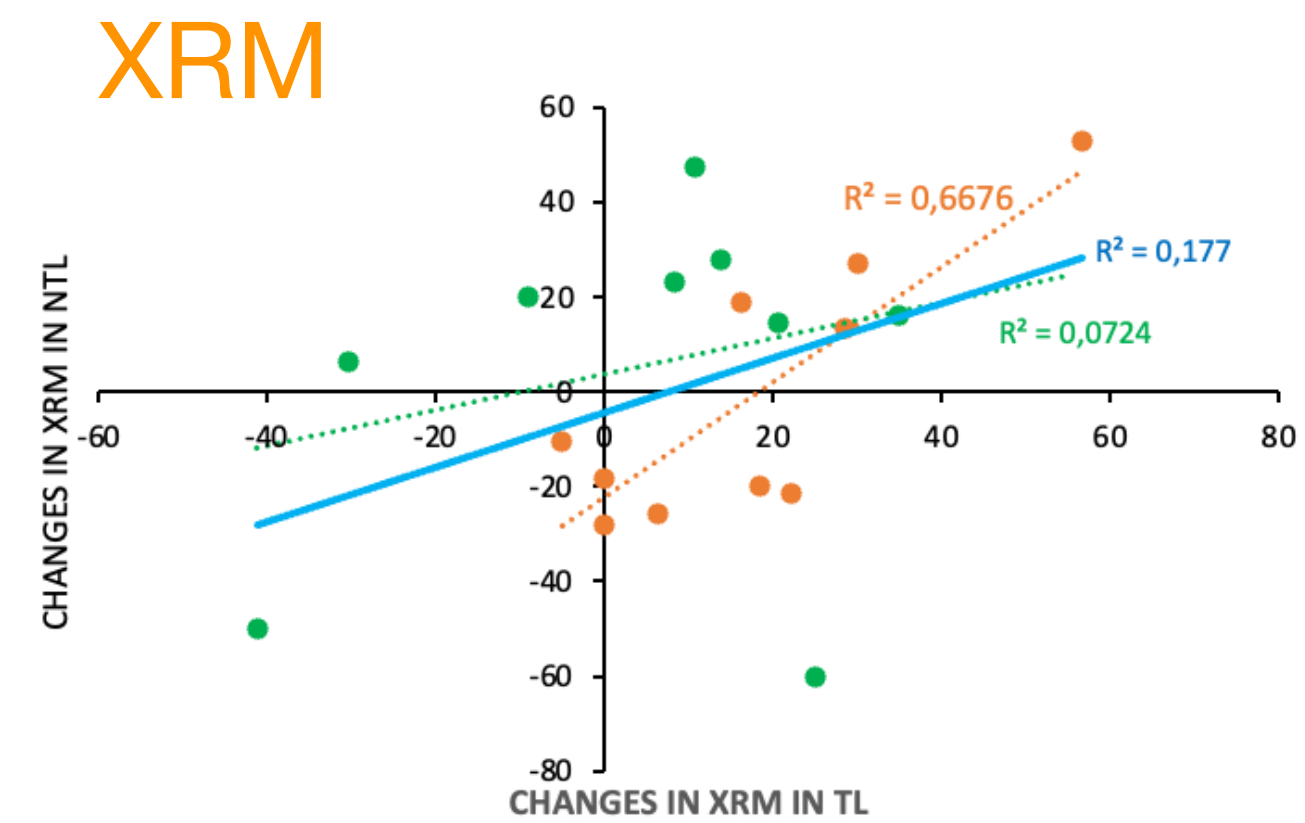

Supplement: Supplementary file 1 [file jfmk-11-00063-s001.zip › jfmk-4005074-supplementary file S1.pdf]
